# Quantification of indocyanine-green-enhanced fluorescence with spectrophotometry (O2C®) in low anterior rectal resection: A prospective study

**DOI:** 10.1007/s10151-024-03062-7

**Published:** 2024-12-12

**Authors:** I. Darwich, S. Demirel-Darwich, C. Weiss, F. Willeke

**Affiliations:** 1 Department of Surgery, St. Marienkrankenhaus Siegen, Kampenstr. 51, 57072 Siegen, Germany; 2 Department of Medical Statistics, Biomathematics, and Information Processing at the University Medical Center Mannheim, University of Heidelberg, Theodor-Kutzer-Ufer 1-3, 68167 Mannheim, Germany

**Keywords:** Bowel perfusion, Low anterior resection, O2C®, ICG, Fluorescence, Colic marginal artery, Anastomotic leak, Cold steel test

## Abstract

**Introduction:**

Despite spectacular visuals and the seemingly convincing rationale of using indocyanine-green-enhanced fluorescence in assessing bowel perfusion during colorectal resections, a lingering sense of subjectivity remains in the challenge of quantifying this fluorescence. This prospective study analyzed the application of O2C® spectrophotometry to quantify zones of fluorescence on the large bowel during low anterior resection.

**Materials and methods:**

Patients receiving a low anterior resection for cancer of the mid- and lower rectum were enrolled in this observational prospective study between February 2020 and December 2022. O2C® blood-flow measurement was performed at three different zones of fluorescence intensity (optimal [O], sufficient [S], and absent [A]), visualized at the designated and already skeletonized site of colon transection. The primary end point was to assess whether the O2C® flow value exceeds 164 arbitrary units (AU) at the zone of optimal fluorescence. The secondary objective was to assess whether there were statistically significant differences in flow parameters between the three zones, thus confirming reproducibility of measurements.

**Results:**

A total of 40 patients were enrolled in this study. Of these, 38 patients remained for statistical analysis with regard to O2C® measurement of the fluorescence zones. The O2C® flow parameter measured at the zone of optimal fluorescence was greater than 164 AU in all cases (100%, *p* < 0.0001). There were statistically significant differences in flow parameters measured at the three different zones of fluorescence (O-S: *p* < 0.0001; O-A: *p* < 0.0001; S-A: *p* = 0.0023).

**Conclusion:**

This study proves the feasibility and reproducibility of quantifying zones of indocyanine green (ICG)-enhanced fluorescence on the bowel. All O2C® flow measurements that were collected at the zone of optimal fluorescence exceeded 164 AU, thereby adding more evidence to this value as a suggested cut-off parameter in terms of bowel perfusion.

## Introduction

In the ongoing search for an objective and practical method to assess bowel perfusion in colorectal surgery, indocyanine-green-enhanced fluorescence angiography (ICG-FA) has emerged as the leading contender over the last decade [1, 2]. Its growing popularity can be attributed to several factors: the impressive imaging that shows clearly delineated zones of tissue ischemia, the ease in its application, and its safety profile [3]. Furthermore, emerging evidence suggests a benefit of ICG-FA in reducing anastomotic leak rates, particularly in low anterior resections [4, 5].

However, the subjective character of fluorescence became evident when some authors attempted to describe its intensity or quality. Gröne et al. described three levels of fluorescence intensity (optimal, sufficient, and absent) on the large bowel at the level of intended transection and eventually the planned site for an anastomosis [6]. Similar observations have consistently been made by our own unit during colorectal resections. This subjectivity in assessing the intensity or quality of fluorescence has prompted efforts to try to quantify it [7]. Currently proposed quantification methods, while promising, still have to find a relevant role in daily surgical practice, partly due to their complexity or impracticality [8].

In a prior investigation, our research team explored bowel perfusion assessment using spectrophotometry and laser Doppler flowmetry with the O2C® device (LEA-Medizintechnik, Giessen, Germany) [9]. The O2C® tissue perfusion measurement regularly generates three different parameters: capillary venous oxygen saturation (SO_2_), relative hemoglobin amount (rHb), and blood flow velocity (flow). The study demonstrated that, among these parameters, only flow consistently and significantly reflected the perfusion status of the bowel. Moreover, logistic regression analysis suggested an O2C® flow value of 164 arbitrary units (AU) as a critical threshold, indicating adequate bowel perfusion and predicting favorable anastomotic healing in low anterior resections.

In the present observational study, we exploited this finding by conducting O2C® measurements of the three different zones of fluorescence intensity at the determined site of transection following colon skeletonization.

This approach intended to serve two main objectives: first, to find out whether it is possible to quantify zones of different fluorescence intensity with O2C® and, second, to check for the reproducibility of these measurements.

## Methods

### Design and perioperative setting

A total of 40 patients with adenocarcinoma of the mid- and lower rectum, scheduled for a low anterior resection, were enrolled in this study if they met the eligibility criteria, including being aged 18 years or older and having a Karnofsky index of 70% or higher. Patients received neoadjuvant chemoradiotherapy (nCRT) or short-course radiotherapy (nRT) if recommended by the multidisciplinary tumor board. Patient data were prospectively collected and entered into a dedicated registry. All patients signed written informed consent. The study protocol received approval from the ethics committee of the Mannheim Medical University (Approval No. 2019-417 M-§ 23b MPG). This study adheres to the Strengthening the Reporting of Observational Studies in Epidemiology (STROBE) guidelines.

All patients received mechanical bowel preparation with oral antibiotics 1 day before surgery, except those with stenosing disease, who received only an enema on surgery day. A prophylactic single-shot intravenous antibiotic was given perioperatively.

### Surgical technique and postoperative follow-up

A laparoscopic low anterior resection (LAR) with total mesorectal excision (TME) was performed. The surgical procedure was performed according to the technical principles described by Heald et al. and Enker et al. [10, 11]. The splenic flexure was taken down, and a high-tie of the inferior mesenteric artery was performed in all cases to achieve maximal colonic mobilization [12, 13]. Following TME and transection of the rectum with a linear endo-stapling device, the bowel was exteriorized via a small incision in the left lower abdominal quadrant. The colon was skeletonized extracorporeally at the designated level of proximal transection and a so-called cold steel test was performed to observe arterial bleeding after severing the colic marginal artery with the cold scissors [14]. If an intersphincteric resection and a colo-anal anastomosis were decided due to a very low rectal cancer, the colon was intracorporeally skeletonized at the designated level of proximal transection before a so-called trans-anal pull-through was performed [15, 16].

A double-stapled side-to-end anastomosis was created unless the patient underwent an intersphincteric resection, in which case a hand-sewn end-to-end anastomosis was carried out. All patients who received an anastomosis were defunctioned with a loop ileostomy.

The anastomotic integrity was assessed with a water-soluble contrast enema in all patients during follow-up, either prior to ileostomy closure or in the case of clinical suspicion of an anastomotic leak. Anastomotic leakage was defined in this study as any presence of extraluminal contrast material, fluid collection, or gas bubbles adjacent to the anastomosis, identified through a computed tomography (CT) scan or a contrast study, regardless of clinical presentation.

### ICG-enhanced fluorescence

**Fig. 1:**
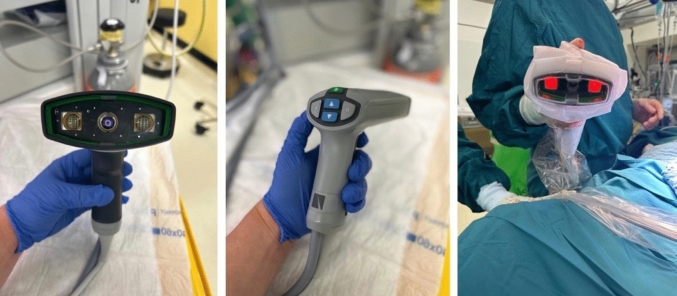
The SPY Portable Handheld Imager (SPY-PHI®, Stryker, Kalamazoo, MI, USA)

**Fig. 2:**
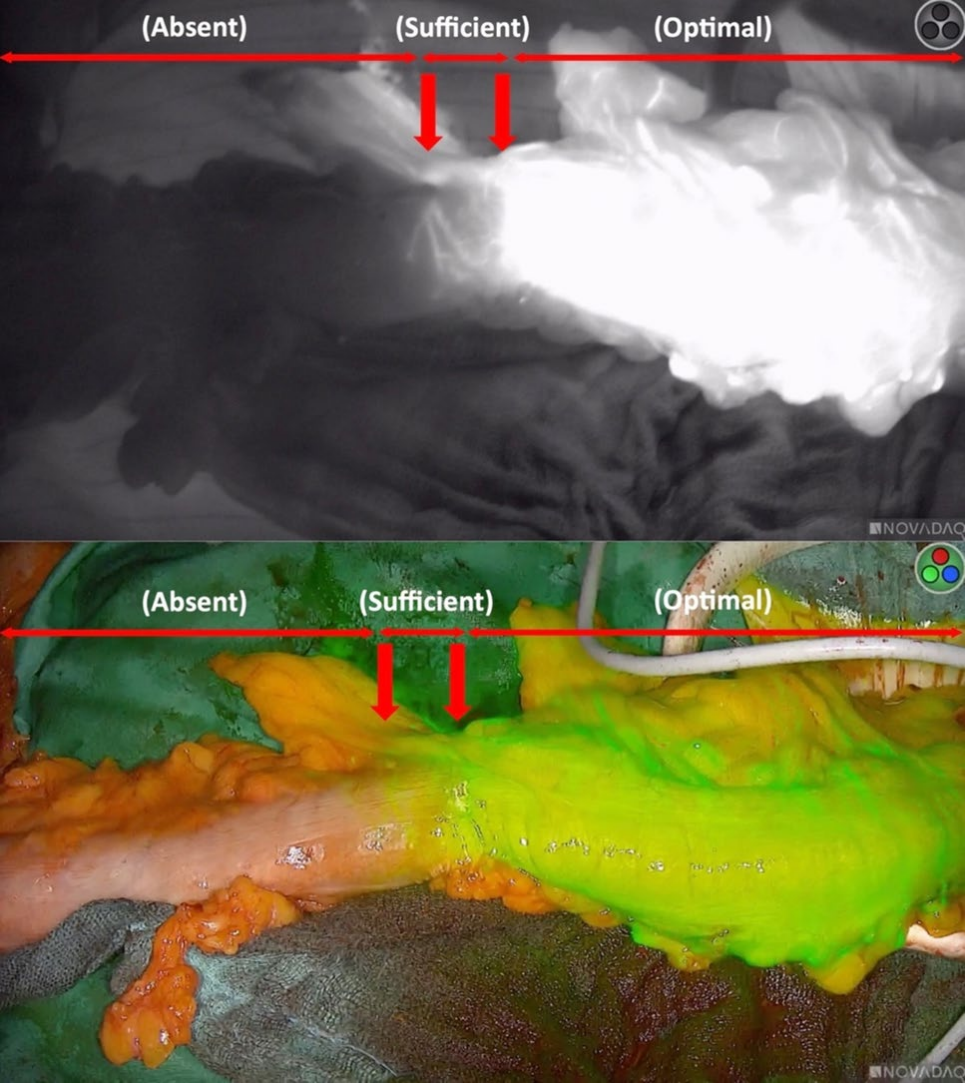
The three zones of different fluorescence intensity as projected by the SPY-PHI®: upper image: spy mode (grayscale); lower image: overlay mode (pseudo-color green)

**Fig. 3:**
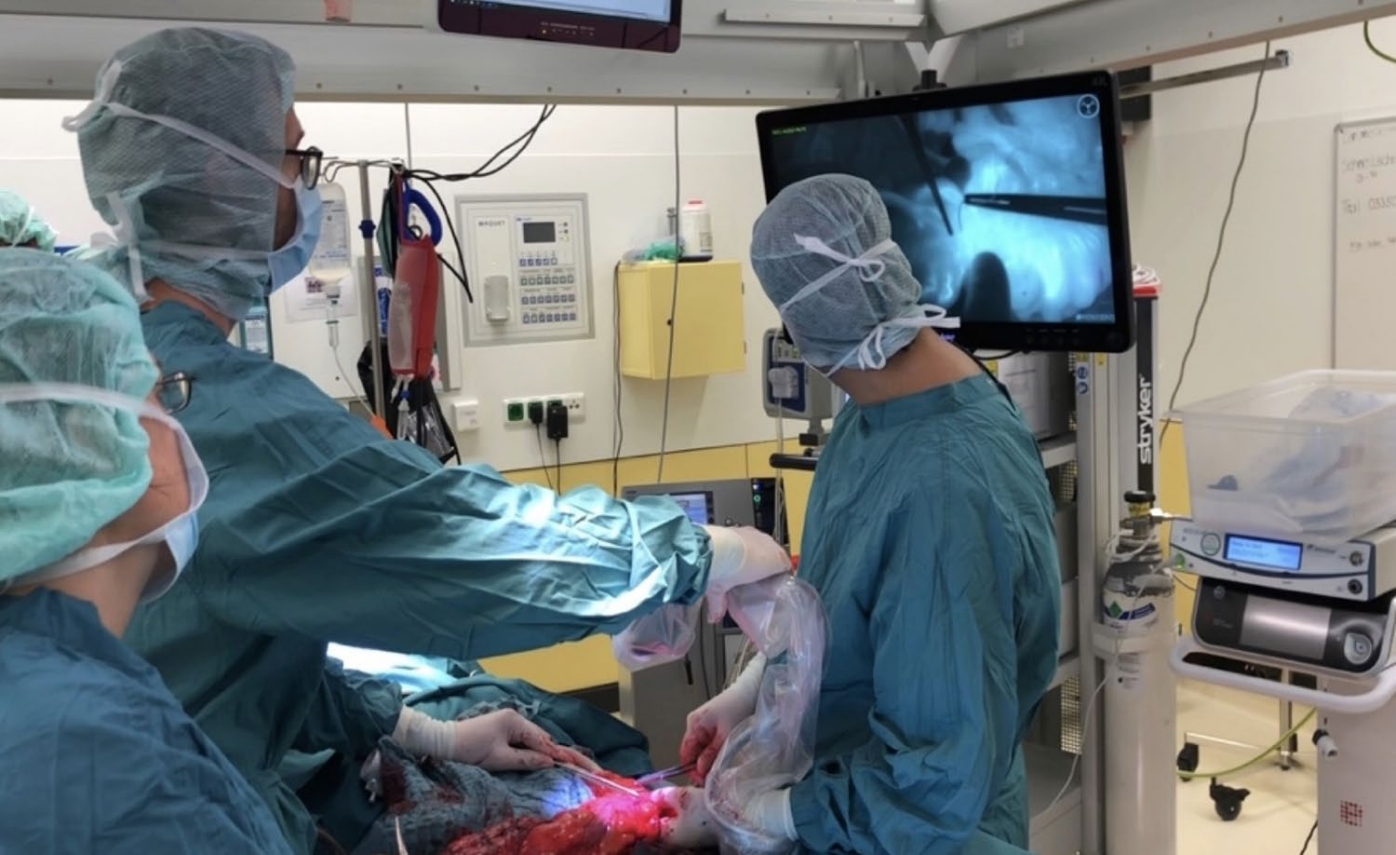
Intraoperative ICG-FA and marking the transition zones with Vicryl stitches

ICG-FA was performed using the sterile-draped SPY Portable Handheld Imager (SPY-PHI®, Stryker, Kalamazoo, MI, USA) to observe the colon under infrared light (Fig. 1) following the intravenous injection of 2 ml (5 mg/ml) of indocyanine green. Two different modes of fluorescence were utilized: (1) overlay mode, where a near-infrared (NIR) image is superimposed in pseudo-color (green) on a white light image, and (2) spy mode, where a NIR fluorescence image is displayed in grayscale. Once the three zones of fluorescence (optimal, sufficient, and absent) had formed (Fig. 2), the transition boundaries were marked (Fig. 3) with 3-0 Vicryl stitches (Ethicon, Inc., Johnson & Johnson, Somerville, NJ, USA).

### O2C measurement

**Fig. 4:**
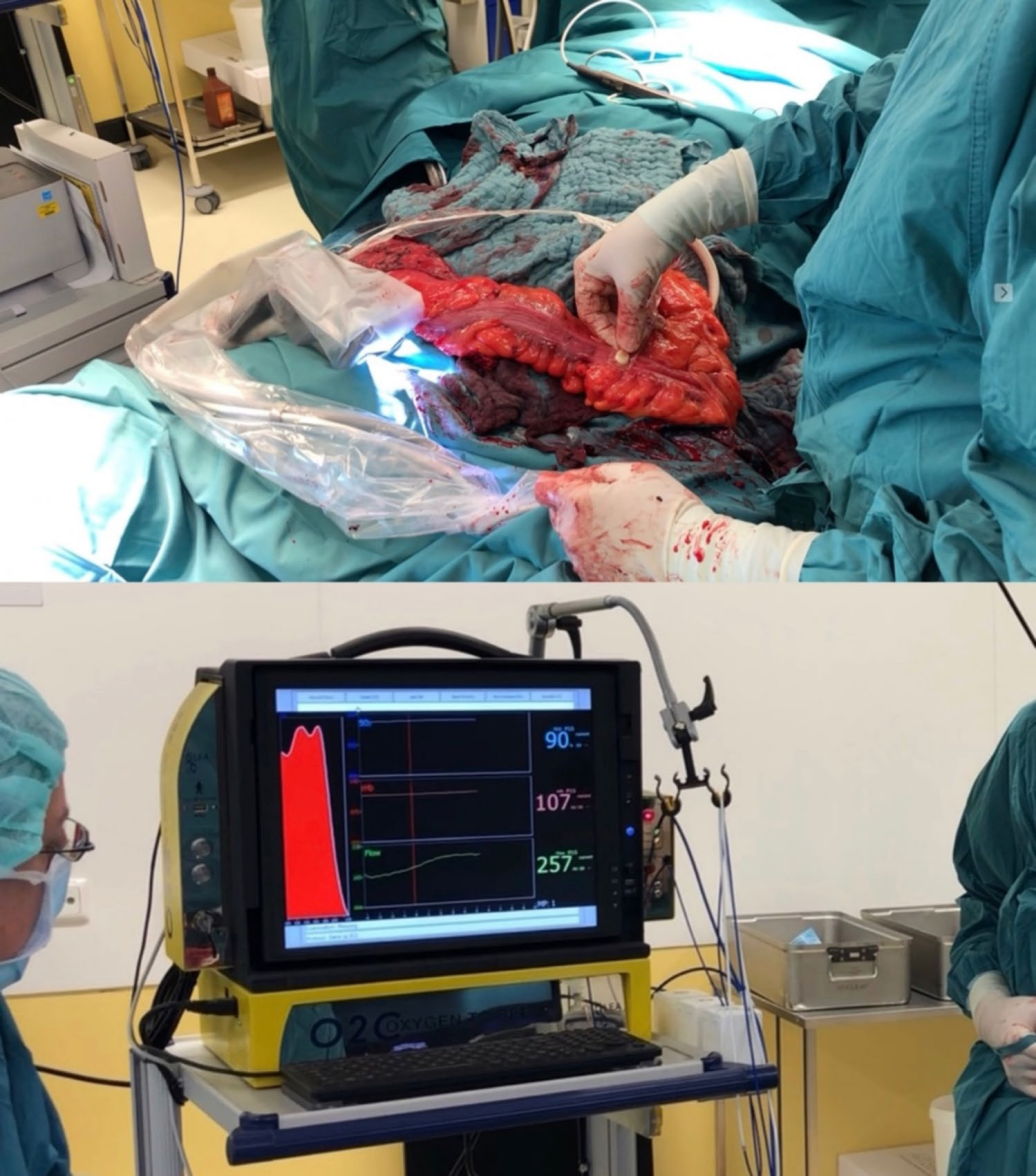
O2C® measurement of the fluorescence zones

**Fig. 5:**
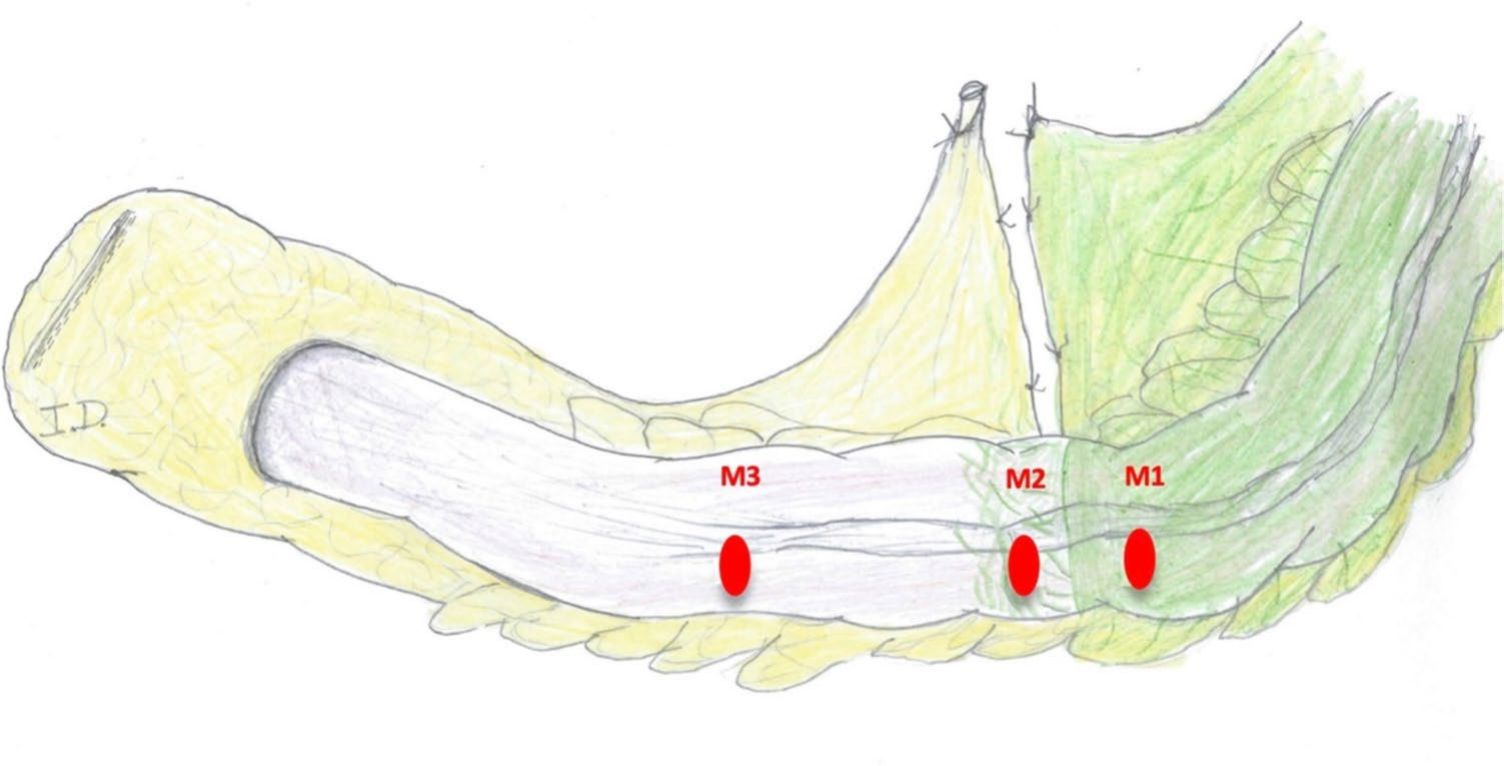
The digital sketch utilized to designate the sites of O2C® measurements at the different zones of fluorescence (M1, M2, and M3)

**Fig. 6:**
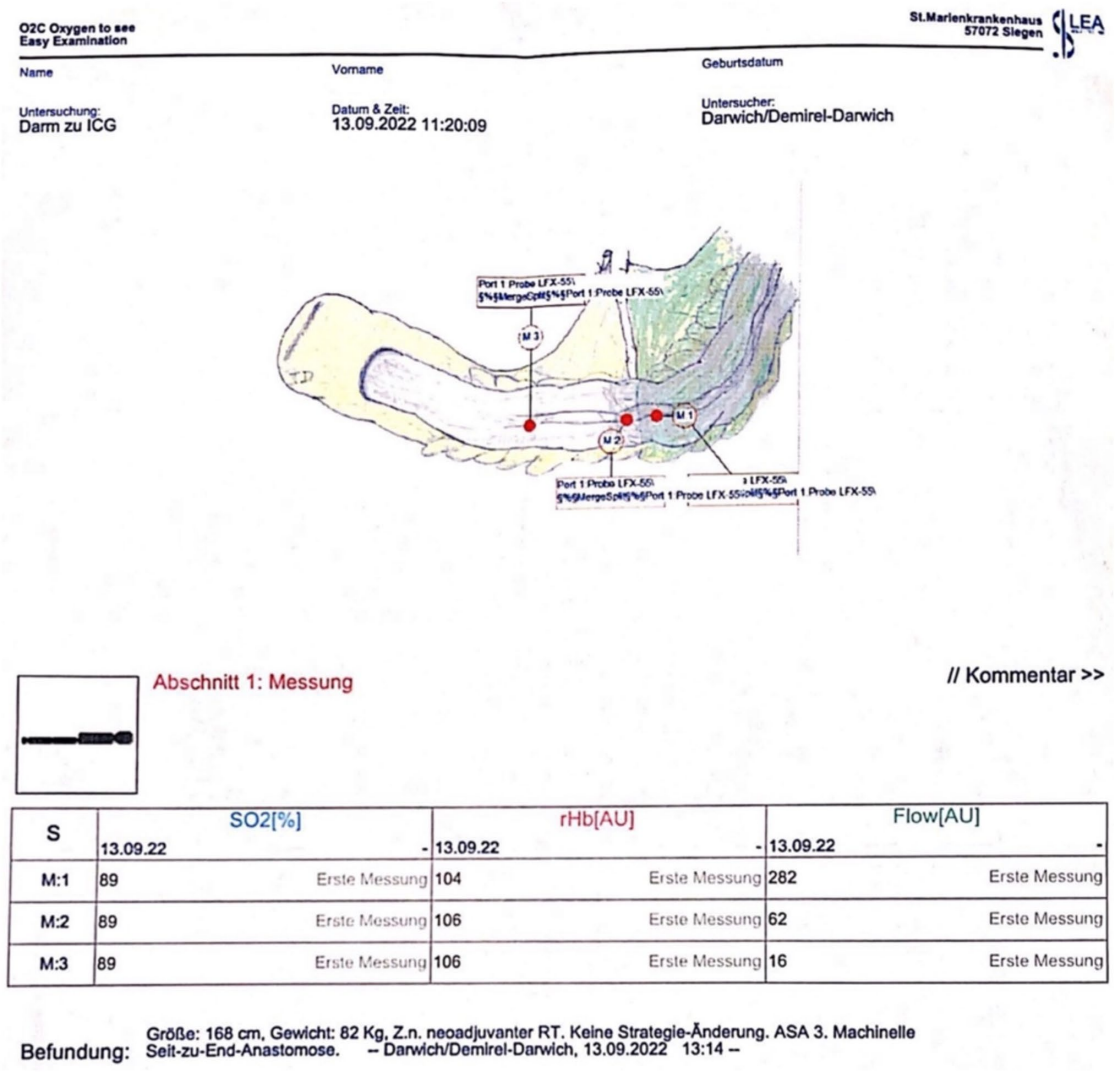
A printed final O2C® measurement protocol

O2C® measurement was then performed at the three marked zones using a flat glass fiber LFX-55 probe (LEA-Medizintechnik, Giessen, Germany), draped with a sterile polyurethane cover (Fig. 4). The measuring technique was identical to that described in a previous study by our group [9]. The O2C® flow parameter (blood flow velocity), expressed in arbitrary units (AU), was measured step-wise at the three different zones (optimal [M1], sufficient [M2], and absent [M3]) of fluorescence (Fig. 5), and the values were saved according to a specially designed protocol that was installed into the device computer (Fig. 6). The saved data included the localization of the measurements, the mode of measurement used, the measured values, and the name of the probe being used. Once the measuring process was accomplished and data were saved, no changes could be made afterwards.

### Study objectives

The primary endpoint of this study was defined as an O2C® flow value, measured at the zone of optimal fluorescence (M1), significantly exceeding 164 AU. The secondary endpoint aimed to detect distinctions in O2C® flow values across the three zones of varying fluorescence intensities, thereby indicating the reproducibility of these measurements. Additional secondary endpoints included the fluorescence-derived adjustment of surgical strategy concerning the level of proximal colon transection, as well as the assessment of associations between fluorescence and other factors in relation to the status of the anastomosis. These included O2C® flow value, measured at the zone of optimal fluorescence (M1) as well as age, sex, body mass index (BMI), American Society of Anesthesiologists (ASA) score, anastomotic technique, and neoadjuvant therapy.

### Statistical analysis

The statistical analysis was performed with the SAS software, release 9.4 (SAS Institute Inc., Cary, North Carolina, USA). For qualitative factors, absolute and relative frequencies are given. Furthermore, 95% exact confidence intervals have been constructed. For quantitative variables, median and range or mean together with standard deviation have been calculated. An analysis of variance for repeated measurements was performed using the SAS procedure “PROC MIXED” to compare the flow values of the measurement locations. Post hoc tests according to Scheffé have been applied for pairwise comparisons (considering patients’ identifier [ID] as a random factor and location as a fixed factor). Associations between anastomotic status and various variables were examined using the Wilcoxon two-sample test, the exact trend test according to Cochran Armitage, or Fisher’s exact test, as appropriate.

The statistical evaluation was carried out by the head of the Department of Medical Statistics, Biomathematics, and Information Processing at the University Medical Center Mannheim, Professor Dr. Christel Weiss.

## Results

Between February 2020 and December 2022, a total of 40 patients were prospectively enrolled in this study (11 female and 29 male). Neoadjuvant therapy was administered to 72.5% of the patients, and all procedures were conducted laparoscopically. The characteristics of the total patient population are demonstrated in Table 1.

**Table 1:** Patient characteristics

Variable	All patients (*N* = 40)
Age (years), median (IQR)	64 (58–71.75); range: 47–93
Male sex, *n* (%)	29 (72.5)
ASA classification, *n* (%) 2 3 4 BMI (kg/m^2^), median (IQR) Neoadjuvant therapy, *n* (%)	19 (47.5) 18 (45.0) 3 (7.5) 27 (24–29); range: 20–41 29 (72.5%)

*ASA* American Society of Anesthesiologists status, *IQR* interquartile range, *BMI* body mass index

**Fig. 7:**
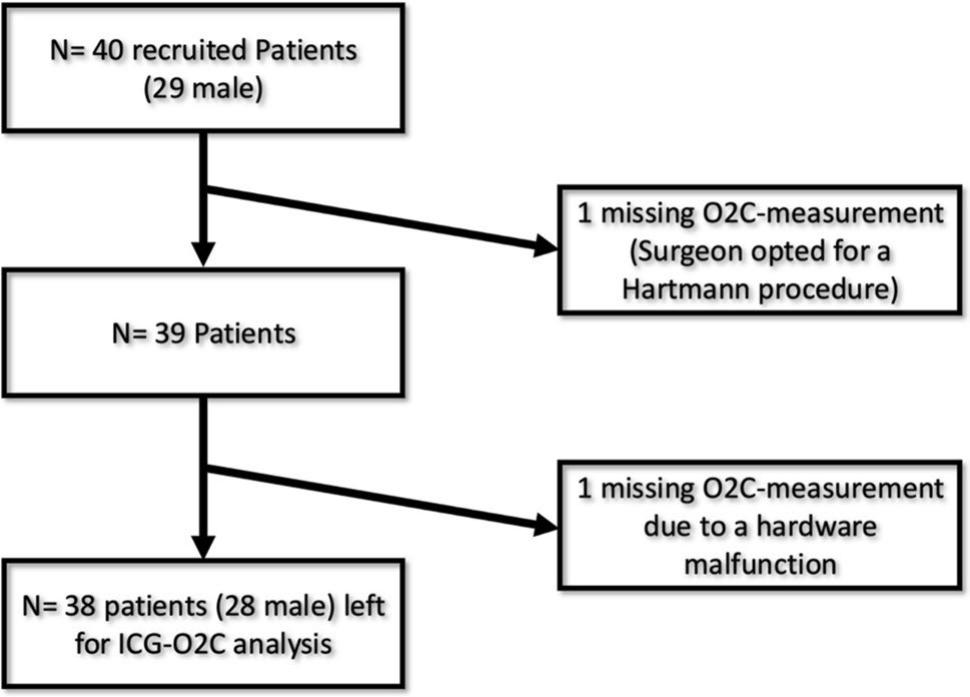
Flowchart indicating patients left for analysis in terms of O2C® quantification of zones of fluorescence

O2C® measurement was omitted by the operating surgeon in one patient undergoing a Hartmann procedure and failed in another patient due to a hardware malfunction. Consequently, 38 patients (10 female and 28 male) remained for statistical analysis regarding O2C® fluorescence quantification (Fig. 7).

From the surgical technical perspective, three patients did not receive an anastomosis. In two of these cases, absence of fluorescence was noted and subsequently attributed to a rudimentary or missing colic marginal artery, necessitating a more proximal transection line on the colon, thus rendering inadequate length for a tension-free colorectal anastomosis and resulting in a Hartmann procedure. Despite the potential for salvage through a Deloyers procedure [17, 18], the surgeon chose to abstain on the basis of personal preference. In the third case, intraoperative frozen section revealed involvement of the distal resection margin, prompting an abdominoperineal resection.

Out of the 37 patients who received an anastomosis, an anastomotic leak occurred in two cases, yielding an overall leak rate of 5.4% in this study (95% confidence interval [CI]: [0.0066, 0.1819]). Fluorescence-influenced surgical decision-making was observed in 2 out of 40 cases (5%, CI: [0.0061, 0.1692]), prompting a shift towards a more proximal line of colon transection.

All O2C® flow values (Table 2) measured at the zone of “optimal” fluorescence (M1) exceeded 164 AU with a confidence interval of 95% [0.9075–1.0000], indicating statistical significance (*p* < 0.0001) when testing the null hypothesis (the probability for exceeding 164 AU equals 50%). Furthermore, 97.4% of O2C® flow values measured at the zone of “sufficient” fluorescence (M2), and all values measured at the zone of “absent” fluorescence (M3) were lower than 164 AU (Table 3), with confidence intervals of 95% [0.8619–0.9993] and [0.9075–1.0000], respectively, both demonstrating statistical significance (*p* < 0.0001).

**Table 2:** Flow values of the patient population (*n* = 38)

Flow	*N*	Mean	Median	Minimum	Maximum	SD
M1	38	244.2	223.5	174.0	502.0	69.1
M2	38	72.4	53.5	14.0	168.0	41.9
M3	38	32.6	26.5	8.0	119.0	23.0

*SD* standard deviation

**Table 3:** Flow values in relation to the threshold of 164 AU (testing H0 [probability > 164 AU = 0.5])

Variable	> 164 AU (*n*)	< 164 AU (*n*)	Procedure	*p*-Value
Mean flow-M1 244.2 AU (range 174.0–502.0)	38	0	FREQ	** < 0.0001**
Mean flow-M2 72.4 AU (range 14.0–168.0)	1	37	FREQ	** < 0.0001**
Mean flow-M3 32.6 AU (range 8.0–119.0)	0	38	FREQ	** < 0.0001**

*FREQ* frequency, *AU* Arbitrary Units

Pairwise comparisons (Table 4) conducted with the Scheffé test revealed notable distinctions among the fluorescence zones. Specifically, significant differences were observed between zones displaying optimal and sufficient fluorescence (*p* < 0.0001 [M1–M2]), optimal fluorescence and absent fluorescence (*p* < 0.0001 [M1–M3]), as well as between zones exhibiting sufficient and absent fluorescence (*p* < 0.0023 [M2–M3]). These results confirm the reproducibility of these measurements.

**Table 4:** Pairwise comparison of the fluorescence zones (*n* = 38)

Difference	Mean ± SD	*t*-Value	Adjusted *p*-Value
M1–M2	171.76 ± 84.4	15.69	** < 0.0001**
M1–M3	211.55 ± 71.7	19.32	** < 0.0001**
M2–M3	39.79 ± 37.6	3.63	**0.0023**

No statistically significant association was observed between the anastomotic status and the O2C® value measured at fluorescence zone M1. Furthermore, factors such as age, sex, BMI, ASA score, anastomotic technique, and neoadjuvant therapy did not significantly impact the anastomotic status (Table 5).

**Table 5:** Analysis of patient characteristics and other variables with respect to anastomotic leak

Variable	Intact anastomosis	Anastomotic leak	Test	*p*–Value
**Mean O2C® flow: M1**			*U*-test	0.7559
247.0 Range: 196–298 AU (*n*)		2		
241.1 Range: 174–502 AU (*n*)	34			
**Sex**			Fisher’s exact test	1.0000
Male *n* (%)	24 (68.6%)	2 (100%)		
Female *n* (%)	11 (31.4%)	0 (0%)		
**Mean age**			*U*-test	0.3294
59.5 years Range: 58–61 (*n*)		2		
65.8 years Range: 47–93 (*n*)	35			
**Mean BMI**			*U*-test	0.6121
26 kg/m^2^ Range: 24–28 (*n*)		2		
27 kg/m^2^ Range: 22–41 (*n*)	35			
**Anastomotic technique**			Fisher’s exact test	1.0000
Hand anastomosis *n* (%)	4 (11.43%)	0 (0%)		
Double-stapled *n* (%)	31 (88.57%)	2 (100%)		
**ASA score**			Cochran–Armitage–Tend-test	0.3063
ASA II *n* (%)	16 (45.71%)	2 (100%)		
ASA III *n* (%)	16 (45.71%)	0 (0%)		
ASA IV *n* (%)	3 (8.57%)	0 (0%)		
**Neoadjuvant therapy**			Fisher’s exact test	0.5120
Primary surgery *n* (%)	10 (28.57%)	1 (50.00%)		
nCRT/nRT *n* (%)	25 (71.43%)	1 (50.00%)		

*n* number of patients

## Discussion

This study evaluated the quantification of three zones of fluorescence on the colon using O2C® spectrophotometry and laser Doppler flowmetry to assess perfusion before creating a colorectal anastomosis. The primary goal was to determine whether O2C® flow values in the optimal fluorescence zone exceeded the cut-off of 164 AU. Secondary objectives included assessing the reproducibility of these measurements.

All O2C® flow values in the optimal zone (M1) exceeded 164 AU (95% CI, 0.9075–1.0000; *p* < 0.0001). In contrast, 97.37% of values in the sufficient zone (M2) and 100% in the absent fluorescence zone (M3) were below 164 AU (*p* < 0.0001). Pairwise comparisons showed significant differences between the optimal and sufficient fluorescence zones (*p* < 0.0001), between the optimal and absent zones (*p* < 0.0001), and between the sufficient and absent zones (*p* = 0.0023), confirming the reproducibility of the measurements. A fluorescence-dependent shift to a more proximal transection line occurred in 5% of cases, leading to two Hartmann resections.

The anastomotic leak rate was 5.4% (2 of 37). Both leaks were successfully managed. No significant association was found between anastomotic status and O2C® flow value at M1, nor with patient factors such as age, sex, BMI, ASA score, anastomotic technique, or neoadjuvant therapy.

Previous studies have explored methods for quantifying ICG-FA, but none have gained significant clinical traction due to the lack of consensus on reliable patterns and standardized protocols [7, 8]. This study’s approach of using O2C® to quantify fluorescence zones is novel. The results confirm the association between optimal fluorescence and O2C® flow values above 164 AU and support the reproducibility of these measurements.

However, the study could not conclusively determine whether an O2C® value > 164 AU predicts uneventful healing, partly due to the small sample size and low leak rate (5.42%). Further randomized studies are obviously needed to clarify this issue.

Meta-analyses [19, 20] have shown that fluorescence reduces anastomotic leak rates in colorectal surgery, though randomized studies such as the FLAG and EssentiAL trials have yielded mixed results [4, 5, 21, 22].

Moreover, the study suggests avoiding anastomoses in the “sufficient” fluorescence zone, as the flow value here was significantly lower than in the optimal zone (72.4 versus 244.2 AU, *p* < 0.0001), despite being higher than the absent fluorescence zone (72.4 versus 32.6 AU, *p* = 0.0023). Though no clear evidence links “sufficient” fluorescence to higher leak rates, caution is advised.

In 5% of cases, fluorescence led to a change in surgical strategy, resulting in Hartmann resections. The 5% strategy change rate was lower than rates reported in previous studies [4, 6, 21], such as Gröne (28%), Alekseev (19%), and De Nardi (11%).

### Limitations of this study and future implications

This study is subject to several limitations. First, the small number of enrolled patients may have influenced the results, particularly evident in the low rate of fluorescence-influenced surgical decision-making and the limited incidence of anastomotic leaks. Second, while the observational methodology and retrospective analysis of prospectively collected data served our initial objectives, randomized studies are necessary to validate these findings. Third, our study does not provide a comparative analysis between ICG-FA and O2C®, but rather elucidates their combined and nearly symbiotic usage. For instance, investigating whether O2C® could effectively replace ICG-FA, especially in scenarios where multiple intraoperative ICG injections impede accurate assessment due to persistent fluorescence from previous applications, would be of considerable interest.

## Conclusion

This study proves the feasibility and practicality of quantifying zones of ICG-enhanced fluorescence on the bowel with O2C®. Furthermore, we successfully demonstrated that bowel segments exhibiting a strong fluorescence pattern consistently register an O2C® flow value surpassing the previously suggested threshold of 164 AU. Additionally, notable variations in O2C® measurements were observed among zones characterized by differing fluorescence intensities, underscoring the reliability and reproducibility of this quantification method.

## Data Availability

No datasets were generated or analyzed during the current study.

## Abbreviations

CI: Confidence interval
ICG: Indocyanine green
ICG-FA: Indocyanine-green-enhanced fluorescence angiography
LAR: Laparoscopic low anterior resection
nCRT: Neoadjuvant chemoradiotherapy
nRT: Neoadjuvant radiotherapy
SD: Standard deviation
TME: Total mesorectal excision

## References

[1] Blanco-Colino R, Espin-Basany E (2018) Intraoperative use of ICG fluorescence imaging to reduce the risk of anastomotic leakage in colorectal surgery: a systematic review and meta-analysis. Tech Coloproctol 22(1):15–23 29230591 10.1007/s10151-017-1731-8

[2] Tang G et al (2022) Effect of indocyanine green fluorescence angiography on anastomotic leakage in patients undergoing colorectal surgery: a meta-analysis of randomized controlled trials and propensity-score-matched studies. Front Surg 9:815753 35372484 10.3389/fsurg.2022.815753 PMC8964518

[3] Safiejko K et al (2022) Safety and efficacy of indocyanine green in colorectal cancer surgery: a systematic review and meta-analysis of 11,047 patients. Cancers (Basel) 14:4 10.3390/cancers14041036 PMC8869881 35205784

[4] Alekseev M et al (2020) A study investigating the perfusion of colorectal anastomoses using fluorescence angiography: results of the FLAG randomized trial. Colorectal Dis 22(9):1147–1153 32189424 10.1111/codi.15037

[5] Watanabe J et al (2023) Blood perfusion assessment by indocyanine green fluorescence imaging for minimally invasive rectal cancer surgery (essential trial): a randomized clinical trial. Ann Surg 278(4):e688–e694 37218517 10.1097/SLA.0000000000005907 PMC10481925

[6] Grone J, Koch D, Kreis ME (2015) Impact of intraoperative microperfusion assessment with Pinpoint Perfusion Imaging on surgical management of laparoscopic low rectal and anorectal anastomoses. Colorectal Dis 17(Suppl 3):22–28 26394739 10.1111/codi.13031

[7] Slooter MD et al (2021) Defining indocyanine green fluorescence to assess anastomotic perfusion during gastrointestinal surgery: systematic review. BJS Open 5:2 10.1093/bjsopen/zraa074 PMC8271268 33893811

[8] Faber RA et al (2023) Quantification of indocyanine green near-infrared fluorescence bowel perfusion assessment in colorectal surgery. Surg Endosc 37(9):6824–6833 37286750 10.1007/s00464-023-10140-8 PMC10462565

[9] Darwich I et al (2019) Spectrophotometric assessment of bowel perfusion during low anterior resection: a prospective study. Updates Surg 8:9 10.1007/s13304-019-00682-9 PMC6892764 31606856

[10] Heald RJ et al (1998) Rectal cancer: the Basingstoke experience of total mesorectal excision, 1978–1997. Arch Surg 133(8):894–899 9711965 10.1001/archsurg.133.8.894

[11] Enker WE et al (1995) Total mesorectal excision in the operative treatment of carcinoma of the rectum. J Am Coll Surg 181(4):335–346 7551328

[12] Rutegard M et al (2012) High tie in anterior resection for rectal cancer confers no increased risk of anastomotic leakage. Br J Surg 99(1):127–132 22038493 10.1002/bjs.7712

[13] Komen N et al (2011) High tie versus low tie in rectal surgery: comparison of anastomotic perfusion. Int J Colorectal Dis 26(8):1075–1078 21445553 10.1007/s00384-011-1188-6 PMC3140934

[14] Novell JR, Lewis AA (1990) Peroperative observation of marginal artery bleeding: a predictor of anastomotic leakage. Br J Surg 77(2):137–138 2317669 10.1002/bjs.1800770206

[15] Swenson O (1950) A new surgical treatment for Hirschsprung’s disease. Surgery 28(2):371–383 15442813

[16] Cutait DE et al (1985) Abdominoperineal endoanal pull-through resection. A comparative study between immediate and delayed colorectal anastomosis. Dis Colon Rectum 28(5):294–299 3996144 10.1007/BF02560425

[17] Deloyers L (1964) Suspension of the right colon permits without exception preservation of the anal sphincter after extensive colectomy of the transverse and left colon (including rectum) technic -indications- immediate and late results. Lyon Chir 60:404–413 14167748

[18] Manceau G et al (2012) Right colon to rectal anastomosis (Deloyers procedure) as a salvage technique for low colorectal or coloanal anastomosis: postoperative and long-term outcomes. Dis Colon Rectum 55(3):363–368 22469806 10.1097/DCR.0b013e3182423f83

[19] Xia S et al (2023) Indocyanine green fluorescence angiography decreases the risk of anastomotic leakage after rectal cancer surgery: a systematic review and meta-analysis. Front Med (Lausanne) 10:1157389 37250631 10.3389/fmed.2023.1157389 PMC10213353

[20] Meyer J et al (2022) Fluorescence angiography likely protects against anastomotic leak in colorectal surgery: a systematic review and meta-analysis of randomised controlled trials. Surg Endosc 36(10):7775–7780 35508666 10.1007/s00464-022-09255-1 PMC9485176

[21] De Nardi P et al (2020) Intraoperative angiography with indocyanine green to assess anastomosis perfusion in patients undergoing laparoscopic colorectal resection: results of a multicenter randomized controlled trial. Surg Endosc 34(1):53–60 30903276 10.1007/s00464-019-06730-0

[22] Meijer RPJ et al (2022) AVOID; a phase III, randomised controlled trial using indocyanine green for the prevention of anastomotic leakage in colorectal surgery. BMJ Open 12(4):e051144 35365509 10.1136/bmjopen-2021-051144 PMC8977759

